# Flammability of Cellulose-Based Fibers and the Effect of Structure of Phosphorus Compounds on Their Flame Retardancy

**DOI:** 10.3390/polym8080293

**Published:** 2016-08-10

**Authors:** Khalifah A. Salmeia, Milijana Jovic, Audrone Ragaisiene, Zaneta Rukuiziene, Rimvydas Milasius, Daiva Mikucioniene, Sabyasachi Gaan

**Affiliations:** 1 Additives and Chemistry, Advanced Fibers, Empa, Swiss Federal Laboratories for Materials Science and Technology, St. Gallen CH-9014, Switzerland; khalifah.salmeia@empa.ch (K.A.S.); milijana.jovic@empa.ch (M.J.); 2 Faculty of Mechanical Engineering and Design, Department of Materials Engineering, Kaunas University of Technology, Kaunas LT-51424, Lithuania; audrone.ragaisiene@ktu.lt (A.R.); zaneta.rukuiziene@ktu.lt (Z.R.); daiva.mikucioniene@ktu.lt (R.M.); rimvydas.milasius@ktu.lt (D.M.)

**Keywords:** cellulose, flame retardant, phosphorus compounds, phosphoramidates, PCFC, TGA

## Abstract

Cellulose fibers are promoted for use in various textile applications due their sustainable nature. Cellulose-based fibers vary considerably in their mechanical and flammability properties depending on their chemical composition. The chemical composition of a cellulose-based fiber is further dependent on their source (i.e., seed, leaf, cane, fruit, wood, bast, and grass). Being organic in nature, cellulose fibers, and their products thereof, pose considerable fire risk. In this work we have compared the flammability properties of cellulose fibers obtained from two different sources (i.e., cotton and peat). Compared to cotton cellulose textiles, peat-based cellulose textiles burn longer with a prominent afterglow which can be attributed to the presence of lignin in its structure. A series of phosphoramidates were synthesized and applied on both cellulose textiles. From thermogravimetric and pyrolysis combustion flow analysis of the treated cellulose, we were able to relate the flame retardant efficacy of the synthesized phosphorus compounds to their chemical structure. The phosphoramidates with methyl phosphoester groups exhibited higher condensed phase flame retardant effects on both types of cellulose textiles investigated in this study. In addition, the bis-phosphoramidates exhibited higher flame retardant efficacy compared to the mono-phosphoramidates.

## 1. Introduction

Cellulose is a natural polymer and exists abundantly in nature as a constituent of plants and microorganisms. Cellulose-based fibers are widely used in the textile industry, filtration, and also for fiber-reinforced composites. Natural cellulosic fibers used in textiles can be obtained from various parts of plants, such as seed (cotton), leaf, cane, fruit, wood, bast (flax, jute), and grass [1]. Though the major chemical composition of these fibers is cellulose, they may also contain other components, such as lignin, hemicellulose, and inorganic salts often identified as ash. The mechanical properties of the fiber are usually attributed to the cellulose components. When required, other components of the fiber can be removed via suitable pretreatments [2]. The burning characteristics of these cellulosic fibers vary considerably and are based on their chemical composition. For example, the presence of a certain quantity of lignin could improve the flame resistance of the fibers [3]. It is reported that a lower concentration of lignin (~10 wt %) in the fiber improves the char formation during thermal decomposition processes [3]. Increased char formation during the burning process is a good indication of flame retardancy. Elsewhere, lignin-rich bast fibers (flax, hemp) have been shown to exhibit a lower heat release rate than leaf fibers (cabuya and abaca). It was concluded that a high content of cellulose relative to other components could increase the flammability of the fibers [1]. However, other researchers have shown that a higher lignin:cellulose ratio (1:2) could lead to lignin-catalyzed depolymerization, which may reduce char yield [4]. 

For certain applications, such as in building, public transportation, and electrical equipment, reaction to fire is a key specification. Use of biocomposites in these sectors requires that their flame retardancy is improved. Natural fibers are an important source of fuels during a fire, but their use in composites can also be advantageous as they can help improve char formation [5,6]. Hence, the pyrolysis and the combustion of natural fibers must be accurately investigated to better understand their contribution to fire. In addition, efficient flame retardant solutions for these natural fibers would further help development of fire-safe bio-based composites.

Cellulose-based fibers owing to the presence of hydroxyl group can be suitably flame retarded with phosphorus based flame retardants [7,8,9,10,11,12]. During the thermal decomposition process the phosphorus based flame retardants can dehydrate cellulose and enhance the formation of char. The flame retardant efficiency of phosphorus compounds can be improved by either having a nitrogen-based external synergist [7,9] or having a nitrogen linked directly to the phosphorus atom (i.e., phosphoramidates) [10,11,13]. It was recently demonstrated that the efficiency of ethyl ester phosphoramidates could be greatly improved by incorporating a hydroxyl-terminating alkyl group linked to the nitrogen atom [13]. 

In this work we have investigated the flammability of cellulose-based textiles obtained from cotton fibers and peat fibers. The flammability of cotton-based fibers is well studied [14]; however, fire-related properties of peat-based cellulose fibers are relatively unknown. Use of biobased fibers for application in composites are increasing and the potential usage of peat-based fibers in such applications are quite interesting as they can be considered renewable, and are cheap and readily available in Europe (especially the Nordic countries) [15]. Peat fibers are obtained from the outermost leaf of the stem of plants like Sheathed Cottonsedge (*Eriophorum vaginatum*), commonly grown in Nordic countries. Though the primary composition of such fibers is cellulose, they may also have lignin, hemicellulose, pectin, fats, minerals, etc., depending on their source [16,17,18].

The first step in this work was to evaluate the flammability of the untreated cellulose textiles using a standard fire test. Knitted cellulose textiles were evaluated for their flammability using the vertical fabric strip, BKZ-VB Swiss standard fire test. To further improve the flammability of cotton cellulose textiles and peat cellulose textiles, we synthesized a series of phosphoramidates with systematic structural variation. These flame retardants (FRs) were applied to the cellulose textiles and the treated cellulose evaluated for their thermal and flammability properties. Thermogravimetric analysis (TGA) and pyrolysis combustion flow calorimeter (PCFC) were used as screening tools to evaluate the efficacy of synthesized phosphoramidates. 

## 2. Materials and Methods 

Cotton cellulose (*Cot-Cell*) and mixed peat cellulose (*P-Cell*) textiles in the form of knitted fabrics were obtained from JVegateksa JSC (Kaunas, Lithuania). As there is no reference literature on the chemical composition of P-Cell textiles, chemical analysis was carried out by an external laboratory (Latvian State Institute of Wood Chemistry, 27 Dzerbenes Str. LV 1006 Riga, Latvia) according to the methods as described in the literature [19]. The Cot-Cell textile was pretreated and of a similar construction to the P-Cell textile (i.e., it was unscoured and had no pretreatments). Both fabrics had been knitted in a single jersey pattern on a 14E gauge one cylinder weft knitting machine; loop length is 11.4 mm, loop density in course direction 4.2 cm^−1^, loop density in wale direction 5.8 cm^−1^ and area density of 492.48 g/m^2^. As-received P-Cell textile was hydrophobic (natural fat from the fibers), it was washed at 90 °C for 30 min with a 2% standard detergent (ECE detergent 98 obtained from Swissatest Testmaterialien AG, St. Gallen, Switzerland). This pretreatment was performed to remove hydrophobic impurities from the fibers and ensure its uniform flame retardant (FR) treatment. As the Cot-Cell fabric was already pretreated and quite hydrophilic, it was used as received in our experiments. All chemicals for the synthesis of the FR compounds were obtained from Sigma Aldrich, Buchs, Switzerland. Unless mentioned, all chemicals used in the synthesis were used without further purification. 

### 2.1. Synthesis of Flame Retardants

The nitrogen-containing phosphoester derivatives synthesized in this work are listed in Table 1. All synthetic procedures involving air- and/or moisture-sensitive compounds were carried out using the standard Schlenk technique under nitrogen atmosphere unless otherwise stated. ^1^H, ^31^P{^1^H} and ^13^C{^1^H} NMR spectra were collected at ambient temperature using Bruker AV-III 400 spectrometer (Bruker Biospin AG, Fällanden, Switzerland). ^1^H and ^13^C chemical shifts (δ) in ppm are calibrated to residual solvent peaks. The ^31^P chemical shifts were referenced to an external sample with neat H_3_PO_4_ at 0.0 ppm.

#### 2.1.1. Synthesis of Mono-Substituted Phosphoramidates (General Procedure)

A solution of dialkyl phosphite (36 mmol) and carbon tetrachloride (39.6 mmol), together with anhydrous THF (40 mL), in a three neck flask was immersed in an ice bath. A mixture of appropriate amine (36 mmol) and triethylamine (36 mmol) in anhydrous THF (10 mL) was then added dropwise under N_2_ atmosphere at a rate such that the temperature did not exceed 10 °C. The resulting mixture was then allowed to warm to ambient temperature and stirred overnight. The mixture was then filtered off and the filtrate was concentrated under reduced pressure. All derivatives (except BA-DEP and BA-DMP) were further purified by vacuum distillation:

**PA-DEP**: bp. 82–85 °C at 0.13 mbar. Yield: 91%, colorless oil. ^1^H NMR (400 MHz, DMSO-d_6_) δ (ppm): 4.82–4.76 (m, 1H) 3.88 (mc, 4H), 2.72–2.64 (m, 2H), 1.39 (*sext*, *J* = 7.3 Hz, 2H) 1.20 (*t*, *J* = 7.1 Hz, 6H), 0.83 (*t*, *J* = 7.4 Hz, 3H). ^13^C{^1^H} NMR (100 MHz, DMSO-d_6_) δ (ppm): 61.01 (*d*, *J* = 5.2 Hz), 42.61 (s), 24.52 (*d*, *J* = 6.0 Hz), 16.13 (*d*, *J* = 6.8 Hz), 11.20 (s). ^31^P{^1^H} NMR (162 MHz, DMSO-d_6_) δ (ppm): 10.0.

**AA-DEP**: bp. 75 °C at 0.35 mbar. Yield: 90%, pale yellow oil. ^1^H NMR (400 MHz, DMSO-d_6_) δ (ppm): 5.82–5.76 (m, 1H), 5.18 (*dq*, *J* = 1.8, 17.2 Hz, 1H), 5.05–99 (m, 2H), 3.89 (mc, 4H), 3.40–33.4 (m, 2H), 1.20 (*t*, *J* = 7.1 Hz, 6H). ^13^C{^1^H} NMR (100 MHz, DMSO-d_6_) δ (ppm): 137.27 (*d*, *J* = 5.5 Hz), 114.5 (s), 61.13 (*d*, *J* = 5.2 Hz), 43.10 (s), 16.09 (*d*, *J* = 6.7 Hz). ^31^P{^1^H} NMR (162 MHz, DMSO-d_6_) δ (ppm): 9.70.

**DA-DEP**: bp. 51–55 °C at 0.30 mbar. Yield: 84%, colorless oil. ^1^H NMR (400 MHz, DMSO-d_6_) δ (ppm): 3.94–3.80 (m, 4H), 3.01–2.93 (m, 4H), 1.21 (*dt*, *J* = 0.6, 7.0 Hz, 6H), 1.02 (*t*, *J* = 7.1 Hz, 6H). ^13^C{^1^H} NMR (100 MHz, DMSO-d_6_) δ (ppm): 61.09 (*d*, *J* = 5.2 Hz), 39.16 (s), 16.06 (*d*, *J* = 6.8 Hz), 14.13 (*d*, *J* = 1.8 Hz). ^31^P{^1^H} NMR (162 MHz, DMSO-d_6_) δ (ppm): 10.15.

**BA-DEP**: was collected as pale yellow oil without any further purification. ^1^H NMR (400 MHz, DMSO-d_6_) δ (ppm): 7 .32–7.20 (m, 5H), 5.47–5.40 (m, 1H), 3.97–3.93 (m, 2H), 3.91–3.79 (m, 4H), 1.16 (*dt*, *J* = 0.4, 7.1 Hz, 6H). ^13^C{^1^H} NMR (100 MHz, DMSO-d_6_) δ (ppm): 140.90 (*d*, *J* = 5.1 Hz), 128.13 (s), 127.14 (s), 126.68 (s), 61.20 (*d*, *J* = 5.3 Hz), 44.31 (s), 16.05 (*d*, *J* = 6.8 Hz). ^31^P{^1^H} NMR (162 MHz, DMSO-d_6_) δ (ppm): 9.68. 

**PA-DMP**: bp. 71–72 °C at 0.32 mbar. Yield: 91%, colorless oil. ^1^H NMR (400 MHz, DMSO-d_6_) δ (ppm): 4.93–4.87 (m, 1H) 3.53 (*d*, *J* = 11.1 Hz, 6H), 2.72–2.64 (m, 2H), 1.39 (*sext*, *J* = 7.3 Hz, 2H) 0.83 (*t*, *J* = 7.4 Hz, 3H). ^13^C {^1^H} NMR (100 MHz, DMSO-d_6_) δ (ppm): 52.17 (*d*, *J* = 5.5 Hz), 42.54 (s), 24.53 (*d*, *J* = 5.5 Hz), 11.16 (s). ^31^P{^1^H} NMR (162 MHz, DMSO-d_6_) δ (ppm): 12.58.

**AA-DMP**: bp. 95 °C at 0.57 mbar. Yield: 93%, colorless oil. ^1^H NMR (400 MHz, DMSO-d_6_) δ (ppm): 5.86–5.76 (m, 1H), 5.18 (*dq*, *J* = 1.8, 17.2, Hz, 1H), 5.12 (mc, 1H), 5.03 (*dq*, *J* = 1.6, 10.3 Hz, 1H), 3.53 (*d*, *J* = 11.1 Hz, 6H), 3.41–3.34 (m, 2H). ^13^C{^1^H} NMR (100 MHz, DMSO-d_6_) δ (ppm): 137.21 (*d*, *J* = 5.2 Hz), 114.71 (s), 52.37 (*d*, *J* = 5.5 Hz), 43.06 (s). ^31^P{^1^H} NMR (162 MHz, DMSO-d_6_) δ (ppm): 12.33.

**DA-DMP**: bp. 41–43 °C at 0.32 mbar. Yield: 85%, colorless oil. ^1^H NMR (400 MHz, DMSO-d_6_) δ (ppm): 3.53 (*d*, *J* = 11.1 Hz, 6H), 2.97 (mc, 4H), 1.03 (*t*, *J* = 7.1 Hz, 6H). ^13^C{^1^H} NMR (100 MHz, DMSO-d_6_) δ (ppm): 52.21 (d, *J* = 5.4 Hz), 39.15 (*d*, *J* = 4.6 Hz), 14.16 (*d*, *J* = 1.7 Hz). ^31^P{^1^H} NMR (162 MHz, DMSO-d_6_) δ (ppm): 12.89.

**BA-DMP**: was collected as pale yellow oil without any further purification. ^1^H NMR (400 MHz, DMSO-d_6_) δ (ppm): 7.33–7.21 (m, 5H), 5.53 (mc, 1H), 3.96 (*dd*, *J* = 7.3, 12.1 Hz, 2H), 3.52 (*d*, *J* = 11.1, 6H). ^13^C{1H} NMR (100 MHz, DMSO-d_6_) δ (ppm): 140.83 (*d*, *J* = 4.9 Hz), 128.21 (s), 127.15 (s), 126.77 (s), 52.38 (*d*, *J* = 5.5 Hz), 44.28(s). ^31^P{^1^H} NMR (162 MHz, DMSO-d_6_) δ (ppm): 12.34.

#### 2.1.2. Synthesis of Bis-Phosphoramidates

##### Synthesis of EDA-DEP

A solution of diethyl phosphite (36 mmol) and CCl_4_ (39.6 mmol) in anhydrous CH_2_Cl_2_ (40 mL) was immersed in an ice bath. A mixture of ethylene diamine (18 mmol) and triethylamine (36 mmol) in anhydrous CH_2_Cl_2_ (10 mL) was then added dropwise under N_2_ atmosphere at a rate such that the temperature did not exceed 10 °C. The resulting mixture was then allowed to warm to ambient temperature and stirred overnight. The volatiles were then completely removed and the product was purified by recrystallization in THF and collected as off-white solid. Yield: 90%, mp. 83 °C. ^1^H NMR (400 MHz, DMSO-d_6_) δ (ppm): 4.88–4.82 (m, 2H), 3.88 (mc, 8H), 2.79–2.74 (m, 4H), 1.21 (*dt*, *J* = 0.4, 7.1 Hz, 12H). ^13^C{^1^H} NMR (100 MHz, DMSO-d_6_) δ (ppm): 61.18 (*d*, *J* = 5.3 Hz), 42.19 (*d*, *J* = 6.0 Hz), 16.08 (*d*, *J* = 6.7 Hz). ^31^P{^1^H} NMR (162 MHz, DMSO-d_6_) δ (ppm): 9.70.

##### Synthesis of EDA-DMP

To a solution of dimethyl phosphite (45.4 mmol) in anhydrous CH_2_Cl_2_ (30 mL), CCl_4_ (45.4 mmol) in CH_2_Cl_2_ (10 mL) was slowly added under N_2_ atmosphere. The solution was then immersed in an ice bath and anhydrous CaCO_3_ (45.4 mmol) was slowly added in small portions. After the complete addition, ethylene diamine (22.7 mmol) in anhydrous CH_2_Cl_2_ (10 mL) was then added dropwise under N_2_ atmosphere in a rate that the temperature did not exceed 10 °C. The resulting mixture was then allowed to warm to ambient temperature and stirred overnight. The reaction mixture was then filtered and the volatiles were completely removed. The product was collected as white solid without any further purification. Yield: 70%, mp. 69 °C. 1H NMR (400 MHz, DMSO-d_6_) δ (ppm): 4.97–4.94 (m, 2H), 3.54 (*d*, *J* = 11.1 Hz, 12H), 2.79–2.74 (m, 4H). ^13^C{^1^H} NMR (100 MHz, DMSO-d_6_) δ (ppm): 52.34 (*d*, *J* = 5.5 Hz), 42.18 (d, *J* = 5.8 Hz). ^31^P{^1^H} NMR (162 MHz, DMSO-d_6_) δ (ppm): 12.30.

### 2.2. Treatments of Flame Retardants and Elemental Analysis

To obtain a certain amount of phosphorus (P) content on cellulose textiles, the Cot-Cell textile and the P-Cell textile were treated with the FR compounds according to the method described in the literature [20]. The required quantity of FR was dissolved in a small quantity of ethanol and poured in a flat shallow beaker. The cellulose textiles were then soaked in this solution and the solvent evaporated by forced air convection. As the FRs had boiling points (BP) higher than the ethanol the solvent evaporated quickly to leave the FRs on treated cellulose. Samples of cellulose were prepared with 2% phosphorus content for each FR. For homogeneity and for reproducible measurements, the cellulose textiles were then cut in small pieces of few millimeters and mixed manually and conditioned for 24 h at 65% RH and 20 °C before any further analytical measurements. The phosphorus (P) content on the treated cellulose samples was measured according to the method described in the literature [20]. Measurements were carried out using the inductively coupled plasma optical emission spectrometry method (ICP-OES), on a Optima 3000(PerkinElmer AG, Rotkreuz, Switzerland) apparatus. Sample preparation for ICP-OES consists of mixing 300 mg of cellulose samples with 1 mL H_2_O_2_ and 3 mL HNO_3_, followed by digestion using a microwave.

### 2.3. Fire Test

BKZ-VB test: The flammability of the knitted cellulose fabrics were evaluated according to Swiss flammability standard (BKZ) with a specific sample size (length: 160 mm, width: 60 mm). In this test, previously conditioned (24 h at 65% RH and 20 °C) cellulose fabrics were placed in a vertical position and subjected to a standardized flame from the lower front edge. The flame height of 20 mm was maintained and should burn constantly with sharp outlines. The burner position was adjusted 45° so that the flame impinges upon the specimen vertically in the middle of the lower edge of the fabric. The flame is brought in contact with the fabric for 15 s and should be placed such that the fabric bottom is initially 4 ± 1 mm inside the flame tip. The test is considered to be passed when two conditions are satisfied: burned length LBV < 150 mm; burning duration tBV < 20 s. The tests were carried out in triplicate and average values are reported. 

### 2.4. Thermal Analysis 

The thermogravimetric analysis (TGA) of the treated cellulose samples was carried out using a TG209 F1 Iris (NETZSCH, Bobingen, Germany). The sample, weighing approximately 4 mg, was heated from 25 to 800 °C at a heating rate of 10 °C/min. The measurements were performed under air or nitrogen atmosphere with a total gas flow of 50 mL/min. 

The combustibility of the treated cellulose was determined by pyrolysis combustion flow calorimetry (PCFC) using a FTT PCFC instrument. The cellulose samples weighing 1–3 mg were pyrolyzed by heating up to 750 °C, at a heating rate of 1 °C·s^−1^. The combustor of the PCFC instrument was maintained at 900 °C. The specific principle of the PCFC analysis is well described in the literature [9,21]. All TGA and PCFC measurements were carried out in triplicate and the average values are reported. 

Py-GC-MS measurements were performed on samples by placing 30–100 µg of the sample which was placed in a quartz tube (1 mm internal diameter × 25 mm length). The polymer was then loaded in the pyrolysis probe (5200 (CDS Analytical Inc., Oxford, PA, USA.) Pyroprobe-2000) and placed in the special inlet at the interface. The P-PI powder was pyrolyzed at 600 °C under helium atmosphere for 30 s. The volatiles were separated by a Hewlett-Packard 5890 Series II gas chromatograph and analyzed by a Hewlett-Packard 5989 Series mass spectrometer. A GC column filled with cross-linked 5% PH-ME siloxane, 0.53 mm diameter and 30 m length, was used. The GC oven was programmed to hold constant at 50 °C for 1 min. The oven was then heated from 50 to 250 °C at a heating rate of 10 °C/min and held at 250 °C for 5 min. 

## 3. Results and Discussion

### 3.1. Choice of Cellulose Materials

As described in earlier section we have chosen two different kinds of cellulose textiles: cotton cellulose (Cot-Cell) and peat cellulose (P-Cell). Cot-Cell and P-Cell are both knitted textiles with similar fabric constructions. For fire tests, the geometry and construction (density and pattern of knit) of textiles play an important role [22,23] in case of fire, and for a reasonable comparison of their burning behavior it is important to keep these parameters constant. An optical image of the knitted P-Cell fabric, its individual components (yarns and fibers) is presented in the Figure S1.

### 3.2. Chemical Composition of Cellulose Textiles 

The chemical composition of the cellulose textiles used in this work are presented in Table 2. A raw cotton cellulose fiber is composed primarily of cellulose and impurities, such as wax (0.4%–1.7%), ash (inorganic salts) (0.7%–1.8%), pectin (0.4%–1.9%), and others (resins, pigments, hemi-cellulose) (1.5%–2.5%) [24]. The majority of these impurities are removed during their pretreatments (scouring and bleaching) [25]. The Cot-Cell textile is composed of only one kind of fiber, whereas the P-Cell textiles are composed of white and brown fibers (shown in Figure S1). As the Cot-Cell textile is pretreated, it is primarily composed of cellulose. The composition of the particular P-Cell textile used in this work, in addition to majority cellulose, has a considerable amount of lignin and hemicellulose (Table 2). No attempt was made to differentiate the chemical composition of the white and brown P-Cell fibers separately using wet chemical analysis. As wet chemical analysis requires tens of grams of materials for analysis, it was not possible to separate the brown fibers (only 15%) from the textile in such quantity.

However, the chemistry of the white and brown P-Cell fibers can be deduced from the Py-GC-MS data as presented in the Figure S2, Tables S1 and S3. It is clear from this data that the primary composition of the white P-Cell fiber (which matches very well with Cot-Cell fibers) is cellulose (Table S2) and the brown P-Cell fiber (Table S3) is composed of cellulose and lignin (phenolic residues as presented in Table S3). Such phenolic residues are typical characteristics of lignin-containing materials [26].

### 3.3. Burning Behavior of Cellulose Textiles and its Thermal Analysis

It is clear from the above discussions that the composition of the P-Cell and Cot-Cell are different and it is important to know if such difference could have impact on their burning behavior. Furthermore, the burning behavior of peat based textiles is not reported in the literature. The burning behavior of Cot-Cell and P-Cell textiles is presented in Table 3. It is noted here that the total burn time for a sample is the sum of its after-flame and after-glow times. Both cellulose-based textiles burn completely, thereby failing the test, however, differences in their burning behavior do exist. The total burning time for P-Cell textiles (~281 s) is longer than that for the Cot-Cell (~199 s). The P-Cell textiles have a longer after-glow compared to the Cot-Cell textiles. The difference could be due to the presence of lignin contained in the brown fibers of the P-Cell. It has been reported that lignin, which is primarily composed of phenolic polymers, burns slowly and during the burning process it could produce aromatic chars [27]. Biomass with higher lignin content has slower pyrolysis rates [28]. Thus, it is clear that, despite the slow burning nature of P-Cell textiles, it is not able to provide adequate level of fire protection by itself. A suitable FR treatment to improve its flame resistance is, thus, needed.

**Figure 1:**
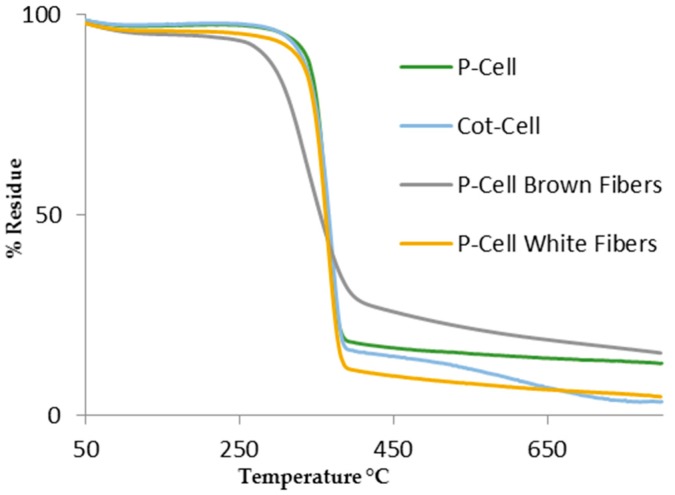
TGA data of cellulose textiles.

Thermal analysis of the cellulose textiles under nitrogen was carried out to further understand their thermal decomposition behavior and gain more insight into their burning characteristics. The TGA curves of the fibers obtained from the cellulose textiles are presented in Figure 1 and the respective data are summarized in Table 4.

To avoid any influence of the shape of cellulose textiles in the thermal analysis, the textiles were cut in very small pieces (2 mm) and the individual fibers obtained were, therefore, homogenously mixed. P-Cell and Cot-Cell in Table 4 represents the data of homogenously-mixed fiber obtained after shredding the textiles. Due to the difference in chemical composition of the fibers of P-Cell textiles, the brown and white fibers were analyzed separately (P-Cell Brown fibers and P-Cell White fibers respectively).

As seen in Figure 1, the weight loss at around 100 °C for all cellulose samples is attributed to moisture [13]. Being hydrophilic, cellulose fibers usually have around 5–10% moisture content. Depending on the chemical composition of the cellulose textiles, all fibers exhibited different peak thermal decomposition temperatures (*T*_dPeak_) and % residue at 800 °C. Cellulose samples containing 100% cellulose (Cot-Cell and P-Cell-White) showed the highest peak decomposition temperature of ~365 °C. Due to the presence of lignin and hemicellulose in the P-Cell-Brown fibers, a reduced *T*_dPeak_ (~348 °C) was observed. A reduced *T*_dPeak_ (~360 °C) for the P-Cell fibers was also observed which is due to its mixed composition (brown and white fibers in a ratio 15:85 wt %, Table 2). The % residue at 800 °C for P-Cell and P-Cell brown fibers is higher than that of the Cot-Cell and P-Cell white fiber. It is known known that cellulosic fibers, being aliphatic in nature, decompose to produce flammable volatiles (primarily levoglucosan) and a very small amount of residue [20]. The presence of lignin in cellulose-based biopolymers increases the char formation which is attributed to possible dehydration of phenolic components of lignin and the eventual formation of thermally-stable char [3,4]. It is very likely that the decomposition of lignin from the P-Cell brown fibers may produce acidic components which can catalyze cellulose dehydration and increase char formation. It has been reported elsewhere that in a cellulose-based material with less than 10% lignin content chemical interaction of lignin and lignin decomposition products with cellulose promotes increased char formation [3].

Combustibility of the cellulose samples (Figure S3 and Table 5) was further carried out using the PCFC instrument to evaluate the peak heat release rate (*Q*_max_) and the total heat to combustion (THR). These values are a good indication of potential fire hazards that these materials pose during their combustion [29]. The PCFC data of the cellulose samples followed a similar trend to that of the TGA data. Due to the presence of lignin, the P-Cell Brown fibers had the highest char formation, lowest heat release rates (~151 W/g), and lowest heat of combustion (~8.5 kJ/g). Thus, the PCFC and TGA data clearly show the difference in the thermal and flame retardant property of the Cot-Cell and P-Cell fibers.

### 3.4. Choice of Phosphoramidates and Application on Cellulose Textiles

**Scheme1:**
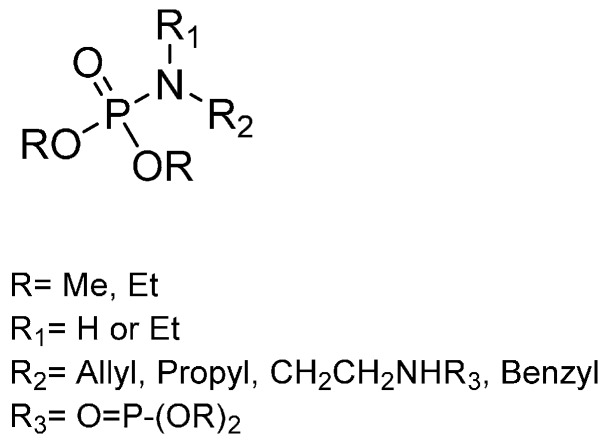
General structure of phosphoester derivatives.

Phosphorus compounds, especially P-N-containing organophosphorus compounds, have been researched extensively as flame retardants for cellulose and other polymers [13,30,31,32,33,34,35,36]. Phosphoramidates have been shown to be efficient in flame retardation of cellulose compared to analogue phosphates [13]. The general structure of all the phosphoramidate compounds synthesized in this work is shown in Scheme 1.

The rationale in synthesizing several derivatives of phosphoramidates was to investigate the difference in the efficacy of methyl vs ethyl phosphester derivatives. The methyl phosphoester derivatives are smaller compared to the ethyl derivatives and also offer sterically less hindrance for a possible nucleophilic attack from nucleophiles during the thermal decomposition process. It is hypothesized such phosphorus compounds work primarily in the condensed phase where a hydroxyl group of cellulose attacks the phosphoryl moiety and displaces the ester or amino functionality of the phosphoester/phosphoramidate group [13]. This is a key step in the condensed phase flame retardant action of the phosphorus compounds in cellulose. The phosphorylation of cellulose retards at an early stage, forming levoglucosan. Levoglucosan is considered the most important fuel precursor of volatiles formed from cellulose decomposition. The presence of various amines on the phosphoramidate moiety offer different degree of steric hindrance. The allyl phosphoramidate (DA-DEP) derivative was of particular interest as it was shown to be very effective in the gas phase flame inhibition [37]. Phosporamidates with different amines linked to an ethyl phosphoester group have been shown to have different flame retardant efficacy [13]. The bis-phosphoramidates (EDA-DMP, EDA-DEP) are relatively large molecules compared to the analogues mono-phosphoramidates used in this study, however, the presence of two phosphorus atoms and the symmetry in their structure makes them the smallest compared to the rest.

The synthesized phosphoramidates were applied to P-Cell and Cot-Cell fabric from ethanolic solutions so as to obtain a theoretical phosphorus (P) content of approximately 2% in the textile. The experimental %P levels of treated cotton cellulose samples are presented in Table 6. 

While attempts were made to prepare cotton cellulose samples with experimental phosphorus compounds close to 2%, there are deviations which may be attributed to incomplete take up of FR solution by the cellulose textiles. Additionally, the variation of the experimental %P was about ±0.2%. 

### 3.5. Thermal and Calorimetry Data of Treated Cellulose Fibers

The flame retardant efficacy of the synthesized phosphoramidates were estimated by evaluating the thermal decomposition characteristics (TGA instrument) and calorimetry (PCFC) data of the treated cellulose textiles. Both methods offer a quick screening method for testing milligram quantities of samples. The TGA curves of the treated cellulose textiles are presented in Figures S3 and S4, and the respective data is summarized in Table 7. All FR-treated cellulose fibers exhibit reduced peak decomposition (main stage) temperatures (~40–70 °C) and enhanced char formation (~10%–30%) compared to the untreated cellulose textiles. This decrease in the main stage of thermal decomposition of cellulose and increased char formation is a clear indication of condensed phase action of the phosphorus-based flame retardants in cellulose [7,9,13]. The effect of structure of phosphoramidate compounds on their condensed phase efficacy is clearly observed from the % residue and *T*_dPeak_ data presented in Table 7. The phosphoramidates with the methyl phosphoester group generally showed higher condensed phase efficacy (increased char formation and reduced main stage of thermal decomposition of cellulose) compared to the ethyl phosphoester analogues. For the same phosphorus compound, the treated P-Cell fibers exhibited higher char formation compared to the Cot-Cell textiles which could be attributed to higher initial char formation (interaction of lignin) for the P-Cell fibers. The structural variation of the amino substituent of the phosphoramidates also had an influence on its condensed phase efficacy. The diamine (bis-phosphoramidates) derivatives (EDA–DEP and EDA-DMP) had the highest condensed phase efficacy compared to the mono-phosphoramidates. It is noted that the difference in the condensed phase activity of the diamino derivatives (methyl vs ethyl phosphoester group) were less compared to the other amines. Among the mono amino derivatives, the benzyl amino and the diethyl amino derivatives exhibited the poorest condensed phase efficacy. As previously reported [13], the thermal decomposition of untreated and treated cellulose textiles showed mostly a three step decomposition: first stage at ~100 °C, corresponds to the release of physically adsorbed water; a rapid second stage at ~360 °C, which corresponds to the dehydration and decarboxylation reactions, producing combustible gasses like aldehydes, ketones, ethers; and the slow third stage at ~400 °C, which corresponds to the decomposition of the char formed in the second stage. In addition to these three stages, a further stage was observed for some amino derivatives (BA-DEP, BA-DMP, DA-DEP, DA-DMP, PA-DEP) from 100–200 °C, which could be attributed to the volatilization of the FR and its decomposition products from the fibers before it can react in the condensed phase with the polymer matrix [13].

To further understand the condensed phase efficacy of the phosphoramidates, PCFC analysis of the treated cellulose samples was carried out. The PCFC curves for Cot-Cell and P-Cell textiles are presented in the Figures S6 and S7, respectively. The summary of PCFC data for the treated cellulose fibers is presented in Table 8 and Table 9. It is clear from the data presented in Table 8 and Table 9 that the total heat of combustion (THR) and the peak heat release rates (*Q*_max_) for all FR-treated cellulose samples are less than the respective untreated cellulose fibers. This data corresponds well with the TGA data of respective cellulose fibers presented earlier. 

In addition, the peak heat release temperature (*T*_max_) and % residue at 900 °C for treated cellulose textiles are also lower compared to the untreated cellulose samples. Lowering of heat release rates and THR usually leads to increases in the respective sample residues. The increased char residue is attributed to carbon from the polymer matrix and may also contain P-N residues [11]. This clearly indicates the condensed phase flame retardant action of the phosphorus compounds. As observed in the TGA data, the phosphoramidates with the methyl phosphoester group exhibited higher flame retardant efficacy compared to the ethyl phosphoester group. Furthermore, the bis-phosphoramidate derivatives showed the best condensed phase effect, exhibiting the lowest THR and *Q*_max_ values compared to the other phosphorus compounds investigated in this work.

In addition to main stage HRR peak, some treated Cot-Cell textiles (AA-DEP, AA-DMP, BA-DEP, BA-DEP) exhibited an additional HRR peak (centered at a lower temperature) which can be attributed to the volatilization of the respective derivatives. In the case of treated P-Cell only BA-DEP and BA-DMP treated P-Cell textiles showed an additional HRR peak. The desripancies in TGA and PCFC data regarding possible volatilization of various FR additives could be due to diffrence in heating rates (10 K/ min in TGA compared to 60 K/ min in PCFC).

### 3.6. FR Structure-FR Efficacy: Possible Mechanism

**Scheme 2:**
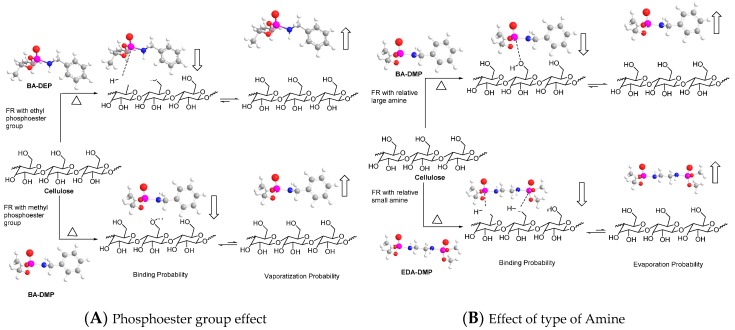
Interactions of phosphorus compounds with cellulose. Different colors.

It can be summarized from the above discussions that the condensed phase activity of phosphoramidates with methyl phosphoester group are higher compared to the ethyl group. Additionally, the bis-phosphoramidates exhibit higher condensed phase activity compared to the mono-phosphoramidates. Phosphoramidates, owing to the phosphoryl groups, interact with the hydroxyl groups of cellulose via hydrogen bonding (as shown in Scheme 2A,B). This hydrogen bonding interaction is further strengthened due to the presence of amine attached to the phosphoryl group, which is able to donate its loan pair electrons in the phosphoryl group. As shown in the scheme, a closer proximity of the hydroxyl groups is possible in the case of phosphoramidates with the methyl phosphoester group compared to the ethyl group. As these phosphorus compounds are not covalently bonded to cellulose, this hydrogen bond interaction may play a very significant role during the thermal decomposition process of cellulose. A closer proximity of phosphoryl groups (less steric hindrance) will favor an efficient and rapid phosphorylation of cellulose compared to the volatilization of the phosphorus compound [13]. The phosphorylation of cellulose in the early stage of thermal decomposition of cellulose is the key step for the effective condensed phase action of the phosphorus compounds [7,9,13]. A less crowded phosphoryl group or a smaller phosphorus compound has more chance of interaction with a cellulose microstructure and is more accessible for the phosphorylation reaction, which happens during the thermal decomposition of cellulose at elevated temperatures. 

In case of bis-phosphoramidate compounds (EDA-DMP), due to the presence of two phosphoryl groups, the interaction of phosphorus compounds with cellulose is higher compared to the mono-phosphoramidate compounds (BA-DMP) (Scheme 2B). Thus, the phosphorylation of cellulose, which is key to the condensed phase activity of the phosphorus compounds, is favored compared to the competing volatilization during the thermal decomposition process. The benzyl group is bulky and may provide additional steric hindrance for the nucleophilic attack of cellulose OH, thereby reducing the rate of the phosphorylation and char formation. The TGA and PCFC data further augments this explanation as benzyl and diethyl amino derivatives (BA-DEP, BA-DMP, DA-DEP, and DA-DMP) were shown to volatilize before the main cellulose decomposition stage (see Figures S4 and S5).

## 4. Conclusions

In this work we have demonstrated that the flammability of the cellulose-based fibers differs depending on their chemical composition. Peat-based cellulose textiles, which contain lignin, burn slowly and produce more char compared to the cotton cellulose textiles. An initial screening of the flame retardancy of these fibers with synthesized phosphorus compounds provides a useful insight into the flame retardant structure-efficacy relationships. Steric hindrance of the phosphorus compounds may be an important factor in the efficient condensed phase activity of the phosphorus compounds. Smaller phosphoester groups exhibit higher condensed phase activity compared to the larger counterparts. Compared to the phosphoester group, the methyl phosphoester groups exhibited higher condensed phase flame retardant effects on both types of cellulose textiles investigated in this study. In addition, the bis-phosphoramidates exhibited higher flame retardant efficacy compared to the mono-phosphoramidates. 

In this work, the flame retardancy of the cellulose materials were indirectly deduced from the small scale fire test (PCFC and TGA experiments). Future efforts will focus on treating the textile fabrics on a larger scale and performing mechanical tests, fire tests (such as Limiting Oxygen Index), and standard vertical and horizontal fire tests. Specific chemical interaction of the flame retardants with cellulose at elevated temperatures will be performed using suitable experimental and modeling techniques. In addition, a potential focus for future investigations would be to covalently crosslink the phosphoramidates with the cellulose and evaluate their durability against common laundry conditions.

## Figures and Tables

**Table 1:**
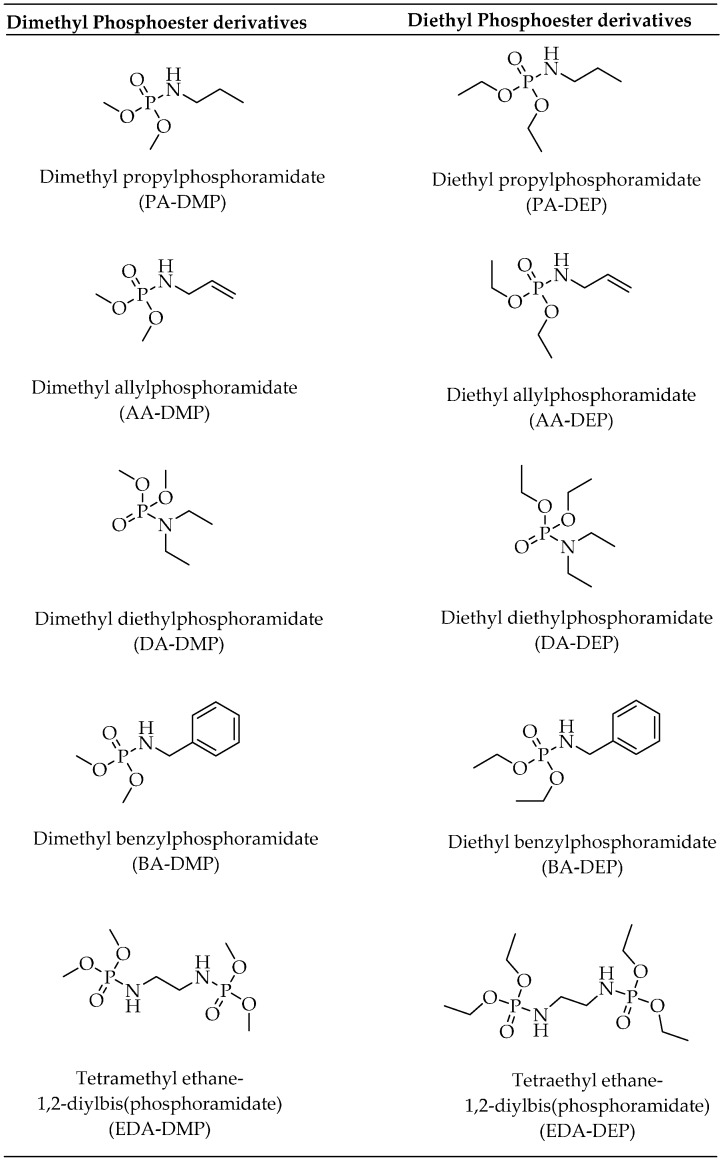
List of synthesized phosphoramidates.

**Table 2:** Composition of cellulose textiles.

Material and description	P-Cell (wt %)		Cot-Cell (wt %)
	Brown fibers	White fibers	
Composition of the textile	15	85	100
Lignin	30	0	0
Hemicellulose	16	0	0
Cellulose	54	100	100

**Table 3:** BKZ-VB fire tests for the knitted cotton textiles.

Sample	After-flame (s)	After-glow (s)	Burn length
Cot-Cell	36.0 (± 1.41)	163.3 (± 15.2)	burns completely
P-Cell	18.6 (± 2.0)	263.3 (± 4.0)	burns completely

**Table 4:** Summary of TGA data of cellulose textiles.

Sample	*T*_dPeak_ °C	% Residue at 800 °C
Cot-Cell	363.7	3.4
P-Cell	360.07	13.00
P-Cell Brown Fiber	348.5	15.57
P-Cell-White Fiber	365.5	4.65

**Table 5:** PCFC data of the cellulose samples.

Sample	THR (kJ/g)	*T*_max_ (°C)	*Q*_max_ (W/g)	% Residue
Cot-Cell	11.8 ± 0.5	370.3 ± 0.9	263.5 ± 5.9	5.6 ± 0.8
P-Cell	11.9 ± 0.1	361.6 ± 10.4	266.3 ± 1.9	5.8 ± 0.6
P-Cell White Fibers	12.1 ± 0.6	366.2 ± 2.0	266.3 ± 1.8	7.6 ± 1.9
P-Cell-Brown Fibers	8.5 ± 0.2	359.5 ± 1.9	151.8 ± 4.1	11.5 ± 4.3

**Table 6:** %P content of treated cellulose textiles.

Cellulose sample	FR type									
	AA-DEP	AA-DMP	BA-DEP	BA-DMP	DA-DEP	DA-DMP	EDA-DEP	EDA-DMP	PA-DEP	PA-DMP
Cot-Cell	2.1	1.7	1.9	2.0	1.8	1.8	2.2	1.7	1.7	2.0
P-Cell	1.8	2.1	1.9	2.0	1.9	1.7	1.9	1.7	2.0	2.2

**Table 7:** TGA data of the treated cellulose textiles.

FR	Cellulose type	*T*_dPeak_ °C	% Residue
Untreated	Cot-Cell	369	3.4
	P-Cell	366	13.0
AA-DEP	Cot-Cell	321	22.2
	P-Cell	296	27.8
AA-DMP	Cot-Cell	299	28.9
	P-Cell	297	31.0
BA-DEP	Cot-Cell	315	14.1
	P-Cell	300	24.6
BA-DMP	Cot-Cell	292	23.5
	P-Cell	292	26.5
DA-DEP	Cot-Cell	319	14.7
	P-Cell	319	23.7
DA-DMP	Cot-Cell	317	15.7
	P-Cell	318	26.3
EDA-DEP	Cot-Cell	293	27.1
	P-Cell	290	30.4
EDA-DMP	Cot-Cell	285	34.3
	P-Cell	292	33.5
PA-DEP	Cot-Cell	298	23.0
	P-Cell	295	25.4
PA-DMP	Cot-Cell	289	31.5
	P-Cell	290	32.5

**Table 8:** PCFC data for Cot-Cell treated with various FRs.

Sample	THR (kJ/g)	*T*_init_ (°C)	*T*_max_ (°C)	*Q*_int_ (W/g)	*Q*_max_ (W/g)	% Residue
Untreated	11.0 ± 0.2		370.3 ± 0.5		256.3 ± 4.8	5.8 ± 0.6
AA-DEP	6.5 ± 0.2	137.6 ± 0.6	319.8 ± 1.3	31.7 ± 4.3	246.5 ± 6.9	12.54 ± 1.6
AA-DMP	4.2 ± 0.5	136.0 ± 7.9	291.4 ± 0.7	8.5 ± 2.4	187.9 ± 2.8	25.9 ± 0.5
BA-DEP	10.9 ± 0.2	197.4 ± 2.0	314.0 ± 1.3	70.8 ± 3.2	227.1 ± 3.8	12.2 ± 0.1
BA-DMP	9.7 ± 0.5	188.7 ± 1.0	289.0 ± 0.6	48.0 ± 1.4	170.3 ± 6.3	21.2 ± 0.9
DA-DEP	9.0 ± 0.6		318.4 ± 4.1		262.3 ± 7.5	14.7 ± 3.5
DA-DMP	8.6 ± 1.2		325.3 ± 7.3		250.6 ± 3.3	15.5 ± 3.7
EDA-DEP	4.5 ± 0.5		292.2 ± 4.8		157.5 ± 15.0	26.5 ± 0.9
EDA-DMP	2.8 ± 0.3		277.0 ± 1.4		126.1 ± 4.0	27.5 ± 0.9
PA-DEP	7.2 ± 0.1		304.0 ± 3.1		207.2 ± 2.9	16.7 ± 1.0
PA-DMP	4.5 ± 0.1		283.5 ± 2.4		178.4 ± 5.8	25.9 ± 1.7

**Table 9:** PCFC data for P-Cell treated with various FRs.

Sample	THR (kJ/g)	*T*_init_ (°C)	*T*_max_ (°C)	*Q*_int_ (W/g)	*Q*_max_ (W/g)	% Residue at 900 °C
Untreated	11.9 ± 0.1		361.6 ± 1.8		266.3 ± 9.9	5.6 ± 0.8
AA-DEP	4.9 ± 0.3		289.2 ± 1.7		152.8 ± 7.0	23.8 ± 0.4
AA-DMP	3.8 ± 0.2		287.5 ± 2.1		146.2 ± 2.2	30.0 ± 0.8
BA-DEP	9.3 ± 0.7	183.5 ± 1.9	289.7 ± 1.8	32.5 ± 1.0	169.6 ± 4.3	18.5 ± 0.4
BA-DMP	8.6 ± 0.8	178.9 ± 1.4	283.2 ± 0.7	33.6 ± 1.2	153.6 ± 5.9	27.1 ± 0.4
DA-DEP	9.9 ± 0.7		329.8 ± 0.9		177.7 ± 0.8	12.3 ± 0.5
DA-DMP	9.0 ± 0.5		331.5 ± 1.4		155.6 ± 3.8	14.1 ± 1.5
EDA-DEP	3.6 ± 0.5		285.0 ± 3.3		97.6 ± 5.2	31.2 ± 0.4
EDA-DMP	2.8 ± 0.5		283.2 ± 0.5		124.6 ± 6.1	32.1 ± 1.2
PA-DEP	6.1 ± 0.1		290.8 ± 2.0		176.3 ± 2.0	23.2 ± 0.4
PA-DMP	3.7 ± 0.3		278.5 ± 1.5		133.7 ± 2.3	28.4 ± 0.5

## Supplementary Materials

The following are available online at www.mdpi.com/2073-4360/8/8/293/s1. Figure S1: Optical Image of (A) P-Cell knitted textiles, (B)P-Cell yarns, (C) P-Cell Brown Fibers, (D) P-Cell White Fibers; Figure S2: PY-GC-MS Chromatogram of P-Cell and Cot-Cell Fibers; Figure S3: PCFC data for cellulose textiles; Figure S4: TGA data for FR treated Cot-Cell; Figure S5: TGA data for FR treated P-Cell; Figure S6: PCFC data for FR treated Cot-Cell with phosphoramidates of methyl phosphoester derivatives (A) and ethyl phosphoester derivatives (B); Figure S7: PCFC data for FR treated P-Cell with phosphoramidates of methyl phosphoester derivatives (A) and ethyl phosphoester derivatives (B); Table S1: Major pyrolysis products of P-Cell White Fibers; Table S2: Major pyrolysis products of Cot-Cell Fibers; Table S3: Major pyrolysis products of P-Cell Brown Fibers.
