# Supplementary material for: Flammability of Cellulose-Based Fibers and the Effect of Structure of Phosphorus Compounds on Their Flame Retardancy
Source: Polymers (Basel). 2016 Aug 10;8(8):293. doi: 10.3390/polym8080293 (PMC6431839; doi:10.3390/polym8080293)
Supplement: Supplementary file 1 [file polymers-08-00293-s001.pdf]

Supplementary Materials: Flammability of Cellulose-Based Fibers and the Effect of Structure of Phosphorus Compounds on Their Flame Retardancy

Khalifah A. Salmeia, Milijana Jovic, Audrone Ragaisiene, Zaneta Rukuiziene, Rimvydas Milasius, Daiva Mikucioniene and Sabyasachi Gaan

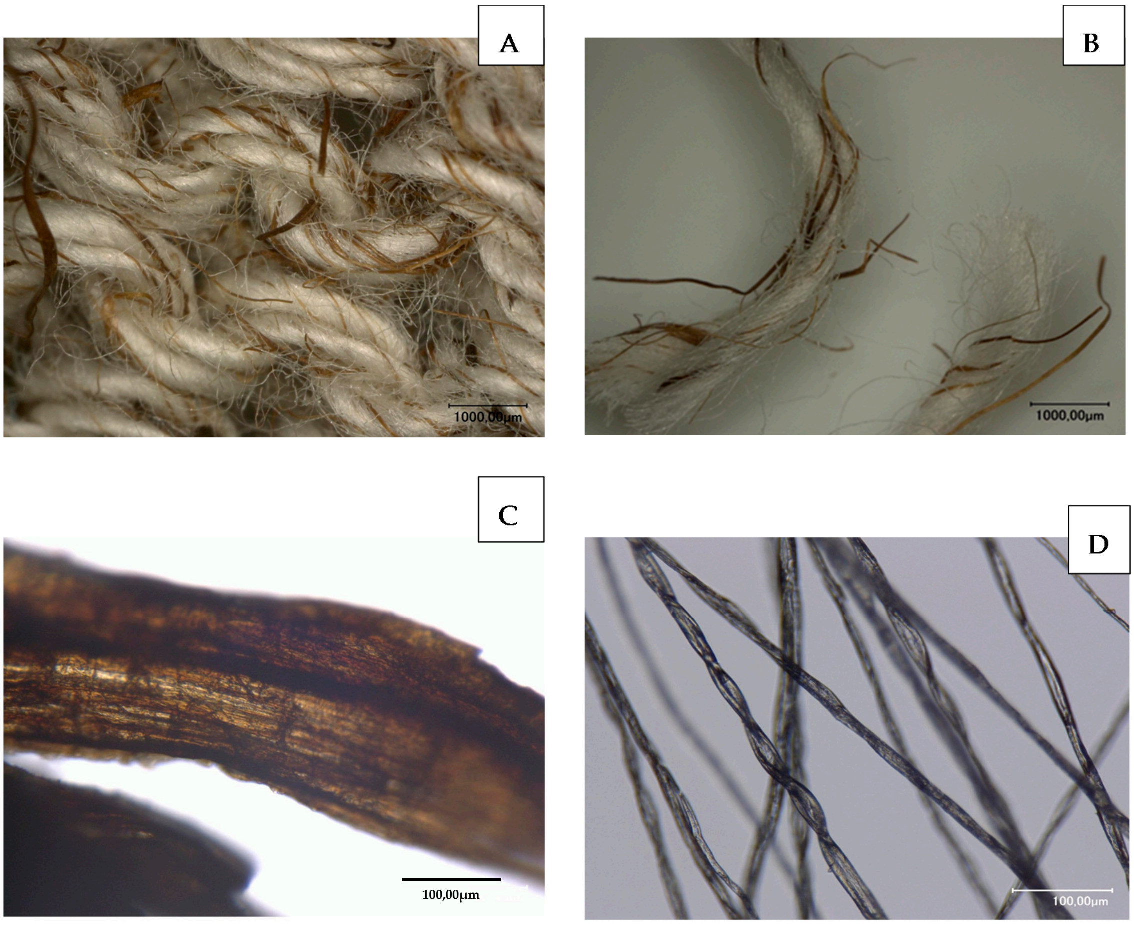

Figure S1. Optical image of (**A**) P-Cell knitted textiles; (**B**) P-Cell yarns; (**C**) P-Cell brown fibers; and (**D**) P-Cell white fibers.

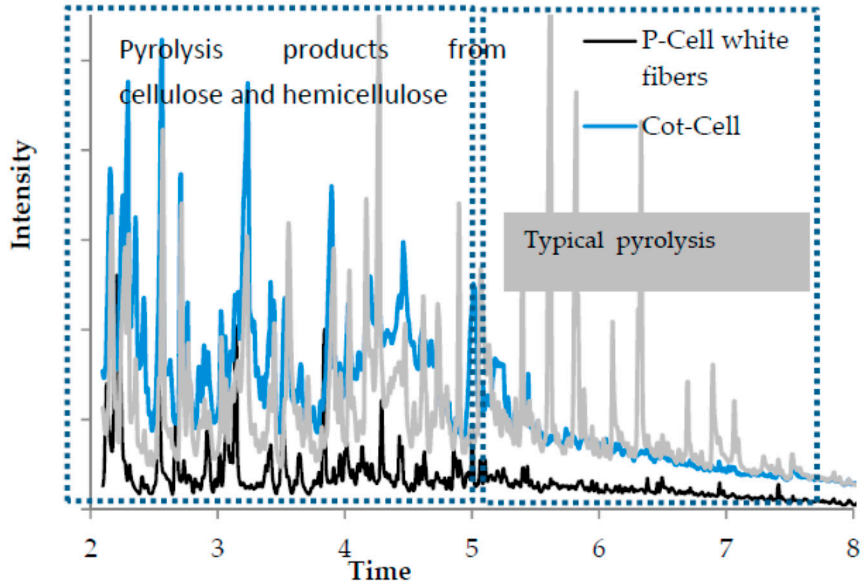

Figure S2. PY-GC-MS chromatogram of P-Cell and Cot-Cell fibers.

Table S1. Major pyrolysis products of P-Cell white fibers.

Peak No	Time, min	Area, %	Pyrolysis products	CAS No.
4	2.539	10.77	Furfural	000098-01-1
6	2,924	5.52	Styrene	000100-42-5
6	2,924	5.52	1,3,5,7-Cyclooctatetraene	000629-20-9
6	2,924	5.52	Bicyclo[4.2.0]octa-1,3,5-triene	000694-87-1
7	3,019	2.25	2-Cyclopenten-1-one, 2-methyl-	001120-73-6
7	3,019	2.25	2,4-Dimethylfuran	003710-43-8
9	3,149	9.17	1,2-Cyclopentanedione	003008-40-0
11	3,517	6.00	Phenol	000108-95-2
11	3,517	6.00	Phosphonic acid, (p-hydroxyphenyl)-	033795-18-5
13	3,843	7.79	1,2-Cyclopentanedione, 3-methyl-	000765-70-8
13	3,843	7.79	2-Cyclopenten-1-one, 2-hydroxy-3	000080-71-7
15	4,139	2.81	p-Cresol	000106-44-5
15	4,139	2.81	Phenol, 3-methyl-	000108-39-4
17	4,430	2.23	2-Cyclopenten-1-one, 3-ethyl-2-h	021835-01-8
19	5,005	1.93	1,4:3,6-Dianhydro-.alpha.-d-gluc	1000098-14-8

Table S2. Major pyrolysis products of Cot-Cell fibers.

Peak No	Time, min	Area,%	Pyrolysis products	CAS No.
9	3.143	5.47	2(5H)-Furanone	000497-23-4
11	3.416	3.80	2-Furancarboxaldehyde, 5-methyl-	000620-02-0
13	3.546	3.29	Phenol	000108-95-2
14	3.896	6.61	1,2-Cyclopentanedione, 3-methyl-	000765-70-8
14	3.896	6.61	2-Cyclopenten-1-one, 2-hydroxy-3	000080-71-7
15	4.026	2.62	Phenol, 2-methyl-	000095-48-7
15	4.026	2.62	Phenol, 3-methyl-	000108-39-4

Table S3. Major pyrolysis products of P-Cell brown fibers.

Peak No	Time,min	Area, %	Pyrolyis products	CAS No.
3	2,716	2.20	2-Furanmethanol	000098-00-0
5	2,870	0.46	Phenol	000108-95-2
6	2,899	0.55	4-Cyclopentene-1,3-dione	000930-60-9
8	3,036	1.02	2-Cyclopenten-1-one, 2-methyl-	001120-73-6
17	3,563	3.82	Phenol	000108-95-2
17	3,563	3.82	Phosphonic acid, (p-hydroxyphenyl)-	033795-18-5
17	3,563	3.82	2-Vinylfuran	001487-18-9
24	3,913	3.25	1,2-Cyclopentanedione, 3-methyl-	000765-70-8
24	3,913	3.25	2-Cyclopenten-1-one, 2-hydroxy-3...	000080-71-7
30	4,174	2.97	p-Cresol	000106-44-5
30	4,174	2.97	Phenol, 3-methyl-	000108-39-4
30	4,174	2.97	Phenol, 2-methyl-	000095-48-7
32	4,275	5.63	Phenol, 2-methoxy-	000090-05-1
32	4,275	5.63	Mequinol	000150-76-5
32	4,275	5.63	2-Acetyl-5-methylfuran	001193-79-9
39	4,618	2.44	Phenol, 2,4-dimethyl-	000105-67-9
39	4,618	2.44	Phenol, 3,5-dimethyl-	000108-68-9
41	4,737	2.13	Phenol, 3-ethyl-	000620-17-7
41	4,737	2.13	Phenol, 4-ethyl-	000123-07-9
41	4,737	2.13	Phenol, 2-ethyl-	000090-00-6
44	4,903	2.27	Creosol	000093-51-6
44	4,903	2.27	2-Methoxy-5-methylphenol	001195-09-1
58	5,620	5.07	2-Methoxy-4-vinylphenol	007786-61-0
62	5,822	4.18	Phenol, 2,6-dimethoxy-	000091-10-1
71	6,331	3.55	Phenol, 2-methoxy-4-(1-propenyl)	005912-86-7
71	6,331	3.55	trans-Isoeugenol	005932-68-3
71	6,331	3.55	Phenol, 2-methoxy-4-(1-propenyl)	000097-54-1

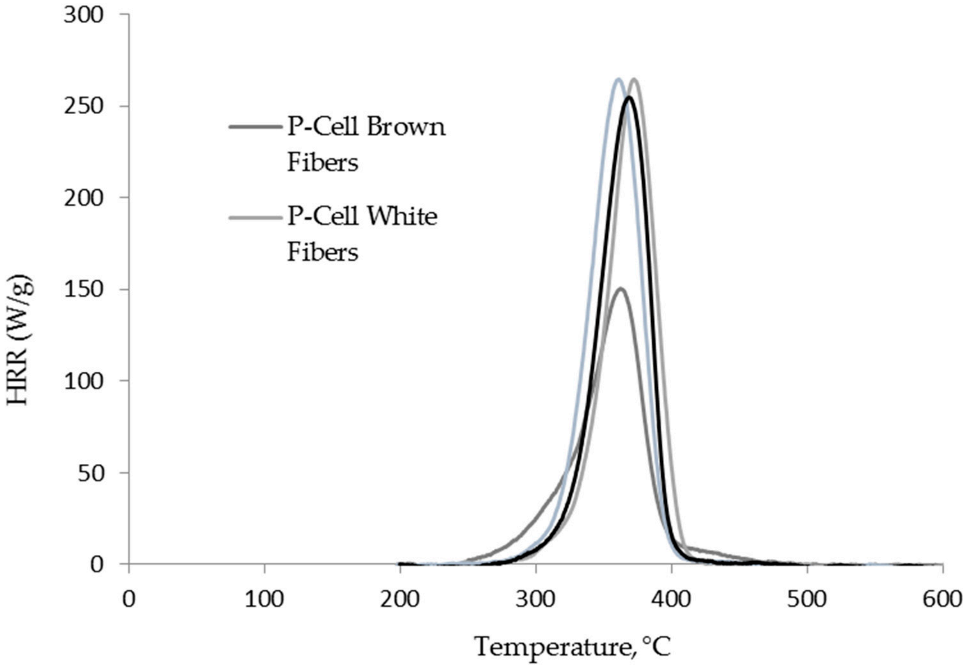

Figure S3. PCFC data for cellulose textiles.

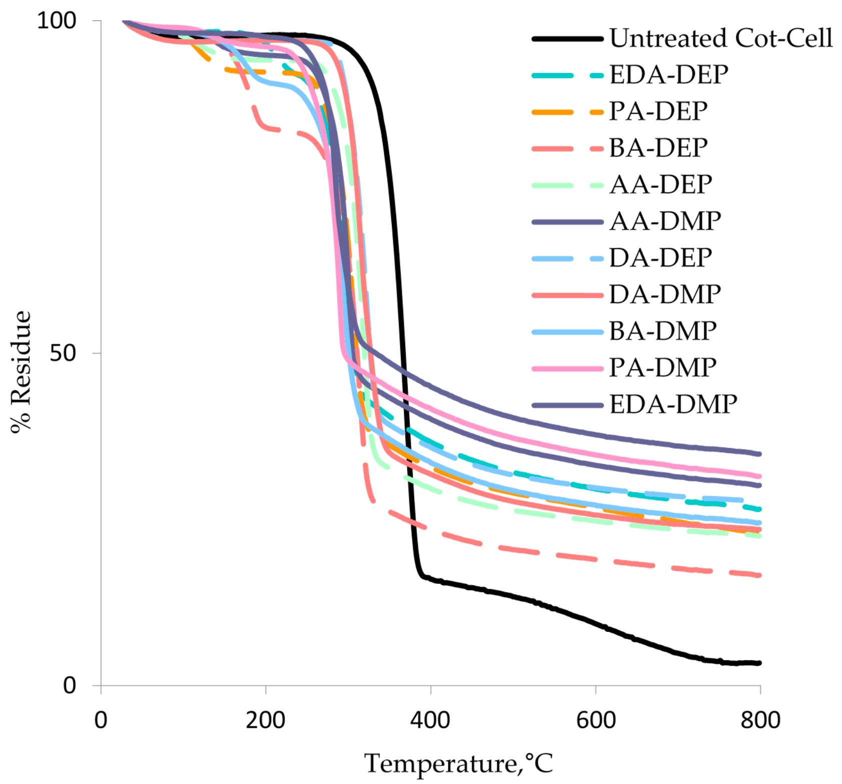

Figure S4. TGA data for FR-treated Cot-Cell.

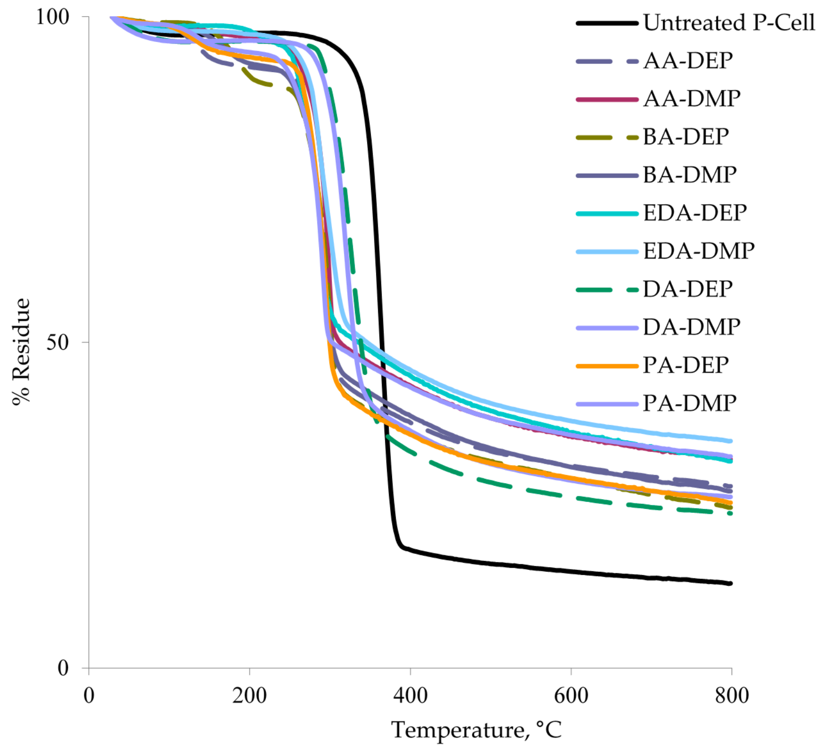

Figure S5. TGA data for FR-treated P-Cell.

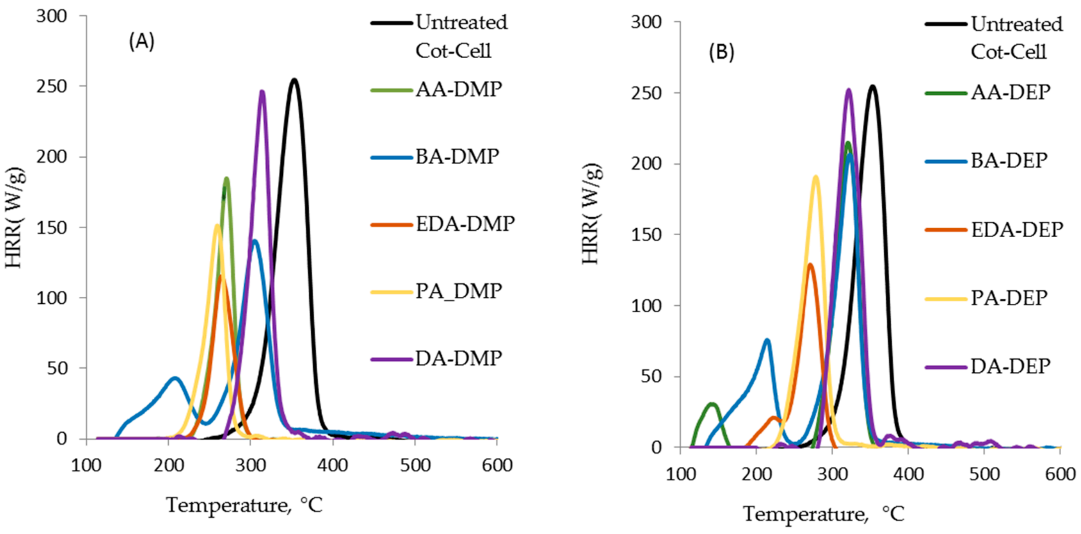

Figure S6. PCFC data for FR-treated Cot-Cell with phosphoramidates of methyl phosphoester derivatives (**A**) and ethyl phosphoester derivatives (**B**).

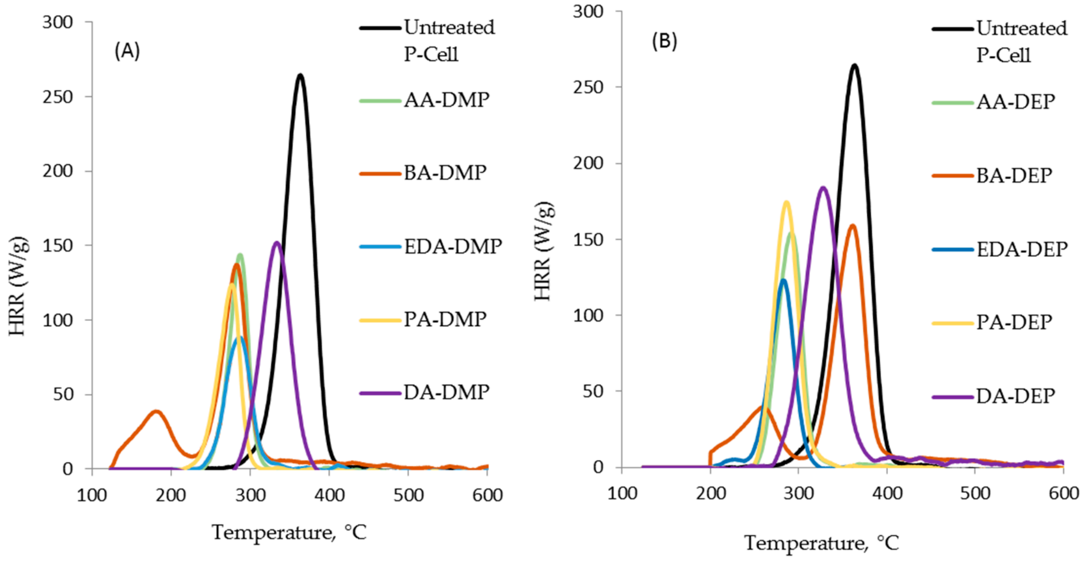

Figure S7. PCFC data for FR-treated P-Cell with phosphoramidates of methyl phosphoester derivatives (**A**) and ethyl phosphoester derivatives (**B**).

© 2016 by the authors; licensee MDPI, Basel, Switzerland. This article is an open access article distributed under the terms and conditions of the Creative Commons Attribution (CC-BY) license (http://creativecommons.org/licenses/by/4.0/).
